# A Population Survey of the Glucose-6-Phosphate Dehydrogenase (G6PD) 563C>T (Mediterranean) Mutation in Afghanistan

**DOI:** 10.1371/journal.pone.0088605

**Published:** 2014-02-21

**Authors:** Natsuda Jamornthanyawat, Ghulam R. Awab, Naowarat Tanomsing, Sasithon Pukrittayakamee, Fazel Yamin, Arjen M. Dondorp, Nicholas P. J. Day, Nicholas J. White, Charles J. Woodrow, Mallika Imwong

**Affiliations:** 1 Department of Clinical Tropical Medicine, Faculty of Tropical Medicine, Mahidol University, Bangkok, Thailand; 2 Mahidol-Oxford Tropical Medicine Research Unit (MORU), Faculty of Tropical Medicine, Mahidol University, Bangkok, Thailand; 3 Department of Molecular Tropical Medicine and Genetics, Faculty of Tropical Medicine, Mahidol University, Bangkok, Thailand; 4 Ministry of Public Health, Islamic Republic of Afghanistan, Kabul, Afghanistan; 5 Centre for Clinical Vaccinology and Tropical Medicine, Churchill Hospital, University of Oxford, Oxford, United Kingdom; Université Pierre et Marie Curie, France

## Abstract

Glucose-6-phosphate dehydrogenase (G6PD) deficiency is a common inherited enzyme defect and an important problem in areas with *Plasmodium vivax* infection because of the risk of haemolysis following administration of primaquine to treat the liver forms of the parasite. We undertook a genotypic survey of 713 male individuals across nine provinces of Afghanistan in which malaria is found, four in the north and five in the east. RFLP typing at nucleotide position 563 detected 40 individuals with the Mediterranean mutation 563C>T, an overall prevalence of 5.6%. This varied according to self-reported ethnicity, with prevalence in the Pashtun/Pashai group of 33/369 (8.9%) compared to 7/344 individuals in the rest of the population (2.0%; p<0.001, Chi-squared test). Multivariate analysis of ethnicity and geographical location indicated an adjusted odds ratio of 3.50 (95% CI 1.36–9.02) for the Pashtun/Pashai group, while location showed only a trend towards higher prevalence in eastern provinces (adjusted odds ratio = 1.73, 0.73–4.13). Testing of known polymorphic markers (1311C>T in exon 11, and C93T in intron XI) in a subset of 82 individuals wild-type at C563 revealed a mixture of 3 haplotypes in the background population and was consistent with data from the 1000 Genomes Project and published studies. By comparison individuals with G6PD deficiency showed a highly skewed haplotype distribution, with 95% showing the CT haplotype, a finding consistent with relatively recent appearance and positive selection of the Mediterranean variant in Afghanistan. Overall, the data confirm that the Mediterranean variant of G6PD is common in many ethnic groups in Afghanistan, indicating that screening for G6PD deficiency is required in all individuals before radical treatment of *P. vivax* with primaquine.

## Introduction

G6PD deficiency is the most common enzyme defect of humans [1]. Many G6PD-deficient individuals remain asymptomatic and unaware of their enzyme deficiency, with no reduction in life expectancy [2]. Clinical manifestations include acute haemolysis following oxidative stress triggered by drugs (such as primaquine, dapsone, chloramphenicol and ciprofloxacin), infection, or the ingestion of fava beans. G6PD deficiency is also associated with an increased risk of neonatal jaundice, and rarely with chronic non-spherocytic haemolytic anaemia and gallstones. The degree of enzyme deficiency and severity of clinical complications depend on the exact G6PD mutation involved. The Mediterranean and certain southeast Asian variants are associated with less than 10% residual enzyme activity (Class II deficiency) [3] and generally more severe clinical manifestations than the African A- form which provides 10–60% of residual activity (Class III). Since the G6PD gene is found on the X-chromosome, there is a higher risk of haemolytic crisis in males (hemizygous) and homozygous females with mutations than in heterozygous females, although heterozygotes are also at some risk due to X-chromosome inactivation [4].

The term ‘Mediterranean’ is used to describe the 563C>T mutation in exon 6 of the human G6PD gene (changing a serine to a phenylalanine residue at position 188 of the protein product), reflecting the first description of this variant in countries such as Italy and Cyprus. However the mutation is also the dominant molecular determinant of G6PD deficiency in many Middle Eastern countries and the Indian subcontinent and has been documented as far east as China [5], Malaysia [6] and Singapore [7]. In south Asia this variant of G6PD deficiency is of practical relevance as there is substantial geographical overlap with malaria. The administration of primaquine to clear the hypnozoite forms of *P. vivax* [8] (radical cure) is compromised by the lack of readily available tests to assess G6PD activity in most locations. G6PD deficiency hence represents a major obstacle to malaria control efforts in Afghanistan and the wider region.

Before commencement of this study, there had been no published studies of G6PD deficiency from within the borders of Afghanistan. The 563C>T mutation has been shown to be the specific molecular cause of most cases of phenotypic G6PD deficiency in two locations in Pakistan [9], [10], and has also been shown to be responsible for the majority of cases of phenotypic G6PD deficiency in Pashtun refugees in Pakistan originating from Afghanistan [11]. G6PD deficiency appears to be at relatively low prevalence in countries north of Afghanistan [12], [13]. It was therefore of interest to investigate the prevalence of the 563C>T allele in Afghanistan itself in both the northern and eastern regions of the country. We also examined two silent polymorphic markers in order to explore the evolutionary history of the Mediterranean mutation in Afghanistan.

## Materials and Methods

### Ethical Approval

Ethical approval for the study was obtained from the Ethics Committee of the Faculty of Tropical Medicine, Mahidol University, Thailand and the Institutional Review Board of the Afghanistan National Public Health Institute. Participants or their parents (in the case of children) provided written informed consent to participate in the study.

### Sites

The study was undertaken in one urban centre in each of nine provinces: Jalalabad (Nangarhar province), Mehtherlam (Laghman), Asadabad (Kunar), Maimana (Faryab), Taloqan (Takhar), Imamsahib (Kunduz), Pulikhumri (Baghlan), Pulialam (Lowgar) and the capital, Kabul (Kabul province). Provinces were chosen on the basis of practical accessibility combined with having a high risk of vivax malaria (>1 case/1000 population/year), the exceptions being Kabul and Lowgar which have low transmission [14].

### Samples

Only males were studied given that the problem of haemolysis after primaquine administration more commonly affects males, and determination of haplotypes is relatively straightforward with single X chromosomes. A convenience approach to sampling was used for practical reasons related to security and resources. EDTA blood was obtained from male adults and children attending outpatient medical laboratories or neighbouring vaccine administration clinics in 2009. No individuals with malaria or infants less than two months of age were included so that malaria and neonatal jaundice were excluded from consideration. There is extensive evidence showing that G6PD deficiency is not associated with other commonly encountered medical conditions that might lead to bias in the selection of cases [1], [2], [15], [16]. Participants or their parents provided written informed consent to participate in the study. Volunteers were asked to state their ethnic group. Samples were transferred to filter-paper blood spots and stored in plastic zip-lock bags with silica gel at room temperature before transport to Bangkok for genotyping.

### Genomic DNA Extraction

Genomic DNA was extracted from dried blood spots using the QIAamp® DNA Mini Kit (QIAGEN, Germany), following the manufacturer’s instructions. Eluted genomic DNA samples were frozen at −20°C until amplification via PCR.

### PCR-RFLP for Detection of G6PD Mediterranean Variant

All samples were subjected to PCR-RFLP to assess the Mediterranean variant in exon 6, based on the protocol of Samilchuk et al. [17] with modifications including use of primers F14948 and R15158 at 250 nM, a volume of 2 µl of each genomic DNA template, a final reaction volume of 30 µl containing 20 mM Tris-HCl (pH 8.4), 50 mM KCl, 1 mM MgCl_2_, 125 µM 4-deoxynucleotide triphosphate (dNTPs), and 0.05 units Platinum®*Taq* DNA polymerase (Invitrogen, Brazil). After pre-denaturation at 95°C for 5 min, 45 PCR cycles were undertaken involving denaturation at 94°C for 1 min, annealing at 55°C for 1 min and extension at 72°C for 1 min, with post-extension at 72°C for 7 min, using a MyCycler™ thermal cycler (Bio-Rad Laboratories, U.S.A.). To examine the 563C>T mutation (Mediterranean variant, Table 1), 10 µl of each PCR product was digested with 10 units of the restriction enzyme *Mbo*II (New England Biolabs Inc.) at 37°C for 3 hours and visualized by 2% agarose gel electrophoresis. A product pattern of 104+98+28 bp indicates G6PD Mediterranean mutation, whereas the digestion pattern 202+28 bp indicates wild-type.

**Table 1:** G6PD molecular markers examined in the study.

Mutation	SNP code	Location	Ancestral allele	Derived allele	Amino acid effect
c.563C>T	rs5030868	Exon 6	C	T	S188F (G6PD Med)
c.1311C>T	rs2230037	Exon 11	C	T	Y437Y
IVSXI C93T*	rs2071429	Intron 11	C	T	Non-coding

Numbers refer to the short isoform version of G6PD. *Commonly referred to as T93C, but correctly annotated as C93T in NCBI dbSNP, consistent with 1000 Genomes and *Pan* spp. data. This mutation could also be formally referred to as c.1365-13C>T.

### Polymorphic Markers

In order to study polymorphic markers relevant to the Asian setting (1311C>T [18] and IVSXI C93T [19], Table 1), in all samples with 563C>T and a subset of samples wild-type at C563, PCR and sequencing of exons 11 and 12 including introns 11 and 12 was undertaken using the protocol of Tang et al. [20]; the forward primer was GAAGCCGGGCATGTTCTTCAAC and the reverse CCAGGGCTCAGAGCTTGTG. All amplification fragments spanning exons 11 and 12 were subjected to gel electrophoresis and purified using FavorPrep™ GEL/PCR Purification kit (Favorgen, Austria). Purified PCR products were assessed by gel electrophoresis and PCR products more concentrated than 50 ng/µl were sequenced at Macrogen, Republic of Korea and analysed using BioEdit version 7.1.3.0. These molecular data were supplemented with relevant data on the same markers either published or downloaded from the 1000 Genomes Project [21], using chromosomes as the denominator. A literature review was also undertaken examining the 1311C>T polymorphism in the context of the 563C>T mutation (minimum of 10 563C>T chromosomes per study); in virtually all these datasets the IVSXI C93T polymorphism was not examined.

### Power and Statistical Analysis

Before the study the available evidence indicated that males from the Pashtun ethnic group had a prevalence of G6PD deficiency of around 10%. Assuming an even mixture of Pashtuns and other groups, a sample size of 512 had 90% power to detect an absolute difference of 7% between Pashtuns and other groups (pooled). In order to ensure sufficient numbers in each of these groups, the sampling was extended to more than 700 patients across nine provinces (covering both northern and eastern regions).

Data were stored and statistical tests undertaken in Stata v12.0.

## Results

### Population Sample

**Figure 1:**
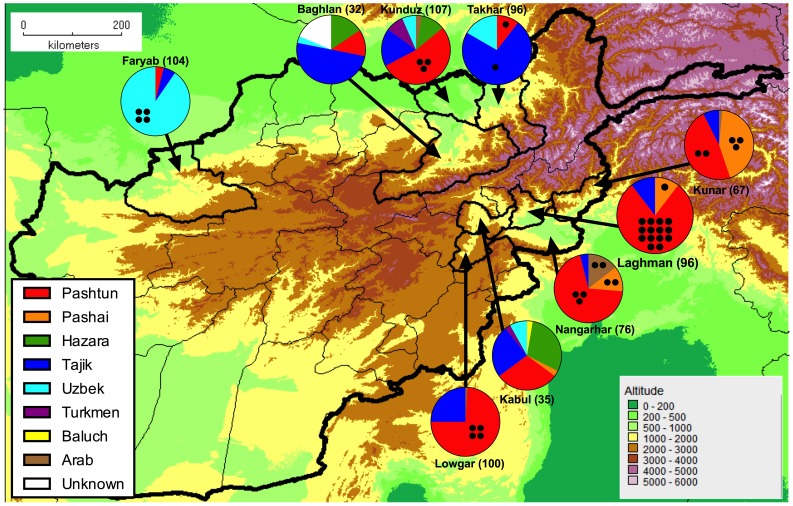
Location, ethnicity and number of Mediterranean variants for the individuals studied. Black circles placed within the relevant segment represent individuals of a particular ethnic group with the 563C>T Mediterranean mutation. The number of individuals sampled in each province is shown next to the province name.

Samples were obtained from 713 individuals across nine provinces of Afghanistan (Table 2, Figure 1), consisting of four in the north (Baghlan, Faryab, Kunduz and Takhar) and five in the east (Kabul, Kunar, Laghman, Lowgar, Nangarhar), these two regions being separated by the Hindu Kush mountain range. There were eight self-reported groups of ethnicity: Arab, Baluch, Hazara, Pashai, Pashtun, Tajik, Turkmen and Uzbek (Table 2) while in 6 individuals ethnicity was not described. As expected, there was a clear link between province and ethnicity with Pashtuns and Pashais generally concentrated in the eastern provinces and Tajiks, Uzbeks and Turkmen generally found in the north.

**Table 2:** Number of individuals with the 563C>T mutation among 713 male individuals studied.

	Ethnicity									
Province	Arab	Baluch	Hazara	Pashai	Pashtun	Tajik	Turkmen	Uzbek	Unknown	Total
Northern										
Baghlan	0	0	0/5	0	0/4	0/16	0	01	0/6	0/32 (0%)
Faryab	0	0	0	0	0/4	0/6	0	4//94	0	4/104 (3.8%)
Kunduz	0/3	0	0/12	0	3/57	0/19	0/9	0/7	0	3/107 (2.8%)
Takhar	0	0	0	0	1/10	1/70	0	0/16	0	2/96 (2.1%)
Eastern										
Kabul	0	0/1	0/10	0/1	0/10	0/9	0/1	0/3	0	0/35 (0%)
Kunar	0/1	0	0	3/29	2/32	0/5	0	0	0	5/67 (7.5%)
Laghman	0	0	0	1/10	14/76	0/10	0	0	0	15/96 (15.6%)
Lowgar	0/1	0	0	0	4/74	0/25	0	0	0	4/100 (4%)
Nangarhar	2/11	0	0	2/9	3/53	0/3	0	0	0	7/76 (9.2%)
**Total**	2/16(12.5%)	0/1(0%)	0/27(0%)	6/49(12.2%)	27/320(8.4%)	1/163 (0.6%)	0/10(0%)	4/121(3.3%)	0/6(0%)	**40/713** **(5.6%)**

The total number of individuals in each province/ethnicity grouping is presented as denominator (zero indicates that there were no individuals present in a given category).

### Mediterranean Variant

The Mediterranean variant (563C>T mutation) of G6PD was found in 40 of the 713 (5.6%) sampled male individuals (Table 2). Prevalence was clearly associated with both province (Pearson chi^2^ = 29.4070, p<0.001) and ethnic group (21.8520, p = 0.005). Grouping the data according to region, the prevalence in northern and eastern provinces was 9/339 (2.7%) and 31/374 (8.3%) respectively. Grouping according to self-reported ethnicity, Pashtuns/Pashais had a G6PD Mediterranean variant prevalence of 33/369 (8.9%) compared to the rest of the population (7/344 = 2.0%). A multivariate analysis undertaken using these methods of grouping indicated a dominant role for ethnicity over geographical location (adjusted odds ratio = 3.50 (95% CI 1.36–9.02) for the Pashtun/Pashai group); location in eastern provinces showed only a trend towards higher prevalence (adjusted odds ratio = 1.73, 0.73–4.13). There was a non-significant trend only towards a lower prevalence in Tajiks compared to Uzbeks (Fisher’s exact p = 0.167).

### Polymorphic Markers and Haplotypes

Two polymorphic markers were examined in all 40 individuals with the Mediterranean mutation and 82 individuals wild-type at C563. These were the synonymous coding sequence polymorphism 1311C>T [18], [22] and a non-coding polymorphism present in the eleventh intron, IVSXI C93T [19]. Both markers are thought to be of no functional significance. Examination of corresponding human 1000 Genomes Project data [23] and available published data from India [24] illustrated that, contrary to many published studies, the ancestral haplotype for the two markers is likely to be C1311/IVSXI C93, (CC haplotype); this is based on the predominance of the CC haplotype in African populations as well as its presence in *Pan* spp.

**Figure 2:**
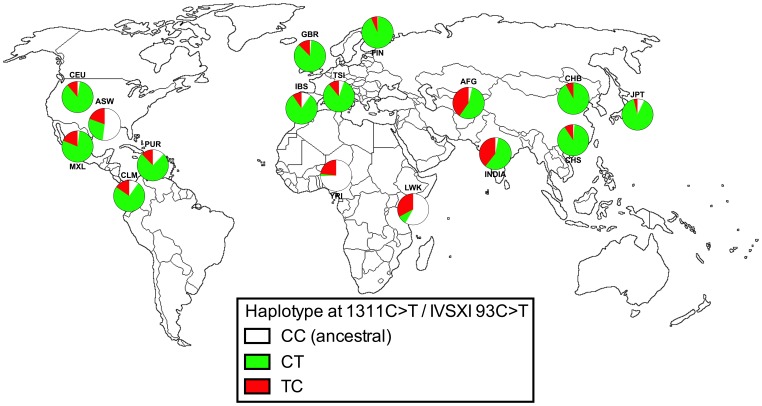
Haplotypes of 1311C>T and IVSXI C93T in G6PD-normal individuals. Data are expressed as total chromosomes and are derived from the 1000 genomes project [23], one published dataset from India [24](INDIA) and this study (AFG). Population abbreviations: ASW, people with African ancestry in Southwest United States; CEU, Utah residents with ancestry from Northern and Western Europe; CHB, Han Chinese in Beijing, China; CHS, Han Chinese South, China; CLM, Colombians in Medellin, Colombia; FIN, Finnish in Finland; GBR, British from England and Scotland, UK; IBS, Iberian populations in Spain; LWK, Luhya in Webuye, Kenya; JPT, Japanese in Tokyo, Japan; MXL, people with Mexican ancestry in Los Angeles, California; PUR, Puerto Ricans in Puerto Rico; TSI, Toscani in Italia; YRI, Yoruba in Ibadan, Nigeria.

The 2-marker haplotypes derived from examination of these positions in Afghanistan are shown in Table 3 and, along with the 1000 Genomes data, in Figure 2. In the 82 C563 individuals (wild-type at the Mediterranean locus) there was a mixture of CT (46) and TC (33) haplotypes, with only 3 ancestral CC haplotypes, consistent with previous data from India [24]. There was no association with ethnic group; among the 38 Pashtun/Pashai individuals studied 1, 21 and 16 had the CC, CT and TC haplotypes respectively, with a very similar pattern in the rest of the population (2, 25 and 17 respectively; p = 0.928 in Fisher’s exact test). Two CT individuals were incidentally found to have a novel synonymous C to T mutation at coding position 1398 in exon 12 (preserving a threonine residue) that is not reported in the 1000 Genomes data or in any publication; this SNP therefore appears so far to be private to Afghanistan.

**Table 3:** Frequency of 1311C>T/IVSXI C93T haplotypes according to allele at 563C>T (Mediterranean).

Allele at 563 Exon 6	Allele at 1311 Exon 11	Allele at IVSXI 93 Intron 11	Number of individuals
C (wild-type)	C	C	3
	C	T	46*
	T	C	33
	T	T	0
T (Med variant)	C	C	0
	C	T	36
	T	C	2
	T	T	0

*Includes 2 individuals with a novel synonymous 1398C>T mutation.

**Figure 3:**
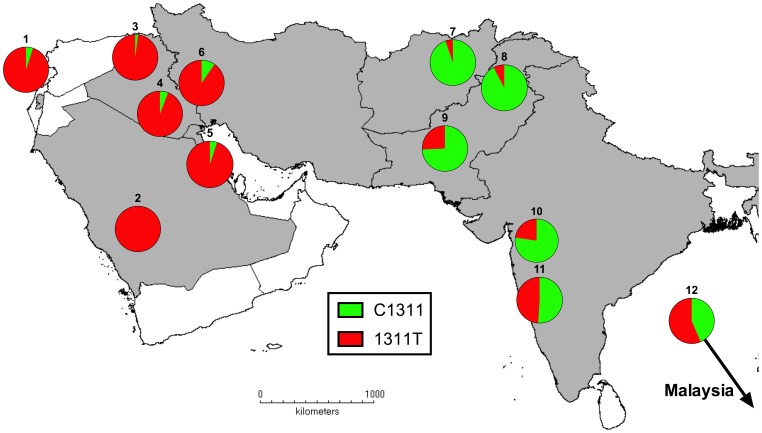
Allele frequencies at 1311C>T marker in individuals carrying 563C>T mutation in southwest Asia. Data are derived from published studies of individuals (expressed as chromosomes) and this study. Countries of study and references are as follows: 1 = Palestine [25], 2 = Saudi Arabia [18], 3 = Iraq (Kurds) [26], 4 = Iraq (Baghdad) [27], 5 = Kuwait [17], 6 = Iran (Kurds) [28], 7 = Afghanistan (this study), 8 = Pakistan (northwest) [10], 9 = Pakistan (Karachi) [30], 10 and 11 = India (Bombay) [29], [31], 12 = Malaysia [6].

In our samples from Afghanistan, the Mediterranean 563T mutation was almost exclusively associated with the CT haplotype (36/38 cases) with only two TC individuals detected; compared to the C563 (wild-type) population this was a highly significant difference (Fisher’s exact p<0.001). We examined these data in the context of relevant published studies (also summarized previously [25]) describing allele frequencies at 1311C>T in individuals with Mediterranean G6PD deficiency in Asia, with work covering the Middle East [17], [25]–[28], the Indian subcontinent [10], [29]–[31] and Malaysia [6](Figure 3). The data obtained here for Afghanistan match those from north-west Pakistan [10] where the wild-type allele (C1311) is the predominant background in Mediterranean variant individuals, in marked contrast to studies from the Middle East where the 563C>T mutation is strongly associated with a 1311C>T background [22].

## Discussion

### Overall Findings

This survey was undertaken primarily in order to assess the prevalence of the Mediterranean G6PD variant in a range of provinces within Afghanistan, spanning regions in the north and east of the country where primaquine is an important part of efforts to treat and control *P. vivax* infection. During this work, an independent study by Leslie et al., taking an analogous approach, but also including phenotypic assessment of G6PD status, was published [32]. The two studies have produced similar findings. Neither study was designed to provide a comprehensive measure of the overall prevalence of G6PD deficiency across Afghanistan, but in both studies the prevalence of the Mediterranean variant 563C>T in males was approximately 5%. In our study the Mediterranean mutation was more common among individuals identifying themselves as Pashtun or Pashai (8.9%); the phenotype-based study reported a prevalence of deficiency of 10.0% in Pashtuns [32]. Individuals reporting themselves to be of Tajik, Uzbek, Hazara or Turkmen ethnicity have lower prevalence of 563C>T in the region of 1–3%. We saw a non-significant trend towards higher prevalence in Uzbeks compared to Tajiks that is consistent with work from within Pakistan [33] and Tajikistan [12], but there was no evidence for this in the other large survey from within Afghanistan [32].

The work reported here involved only genotypic assessment of the Mediterranean allele, and so did not assess whether there are other molecular causes of G6PD deficiency in Afghanistan. In the phenotype-based survey from Afghanistan [32], only 2 of the 44 individuals found to be G6PD deficient by the fluorescent spot test (and successfully genotyped) proved to be wild-type at C563, so it is unlikely that our C563-focused approach missed a significant number of other molecular causes for G6PD deficiency. Nevertheless, a more systematic genotyping strategy might have revealed rare cases of G6PD deficiency with an alternative molecular cause in Afghanistan.

### Population Genetics

A number of polymorphic markers for G6PD have been described and used to explore the evolution of the gene in various contexts, and the abnormal functional variants thought to offer protection against malaria in particular. Several markers are only polymorphic in Africa (with mutation producing the A and A- forms) and only the markers 1311C>T and IVSXI C93T are of relevance in Asia. We noted that in the published literature, this second marker is generally interpreted as a T93C mutation, the reason being a historical one in that the first studies of this polymorphism were undertaken in Asia, where the T allele predominates in this position. However a global analysis of haplotypes indicates that both polymorphisms are ancient mutations that almost certainly predate human migration out of Africa, and the ancestral allele is clearly a C at both positions; it is also labelled as such at the NCBI SNP database. In Eurasian populations the ancestral CC genotype has become rare, and the single mutant IVSXI C93T haplotype (C1311, IVSXI 93T, referred to as CT) predominates, presumably as a result of genetic drift associated with population bottlenecks during human migrations into Europe and Asia. The TC haplotype (mutation at 1311C>T only) is rarely found in the general population in the Mediterranean and the Far East but is found at intermediate levels in Africa, India and (as shown here) Afghanistan (Figure 2). It is unsurprising that the double mutant TT haplotype has never been recorded, since this would have required recombination between two markers separated by approximately 150 DNA base pairs.

Understanding this haplotype background is a useful basis for exploring the history of the Mediterranean variant in Europe and Asia. The 563C>T mutation is likely to have been relatively recently introduced into southern Europe/Middle East [34] where it is strongly associated with the 1311C>T mutation (as far east as Iran and Iraq). In contrast, our study found a strong predominance of the wild-type allele at position C1311 in association (‘hitchhiking’) with the Mediterranean mutation in Afghanistan, findings that are consistent with previous data from Pakistan [9], [10] and India [22], [31]. The data therefore provide further support for the idea of distinct origins for the 563C>T mutation in southern Europe and the Middle East compared to Afghanistan and Pakistan [22], with little admixture between these populations in the subsequent period (despite the relatively small intervening distance).

It is not possible with the data obtained to comment in robust way on the driving forces and timescale for the emergence of the 563C>T Mediterranean allele in Afghanistan; this would require examination of markers over a wider length of the X-chromosome to determine the extent to which homozygosity extends beyond the polymorphic markers already examined [35], and/or a study of nearby microsatellite repeats [34]. The data do show that in individuals in Afghanistan without the Mediterranean mutation, background polymorphic markers are evenly mixed among various ethnic groups, a finding that is consistent with the late development of population structure between Afghanistan’s various ethnic groups after the Bronze age [36]. The relatively high prevalence of 563C>T reached in Pashtuns (8.4%) and the clear differences in its prevalence between different ethnic groups, also described in another large survey [32], are consistent with positive selection occurring after that period, in an analogous manner to the emergence of both the 563C>T variant in Europe and the Middle East [34] and the G6PD Mahidol variant in southeast Asia [37]. The detailed mechanisms by which G6PD deficiency might provide a fitness advantage remains unclear, but whereas *P. falciparum* appears to be a driving force for selection of the A- variant in Africa [38], recent studies in Asian populations indicate that protection against *P. vivax* infection may be more relevant [11], [37].

## Conclusions

This genotypic study of the Mediterranean variant of G6PD deficiency in males in nine provinces in Afghanistan confirms that the 563C>T mutation reaches its highest frequencies in Pashtuns and Pashais (8.9%), with lower prevalence in groups historically associated with northern provinces and countries to the north of Afghanistan. The findings illustrate the need to assess G6PD status in all individuals (irrespective of stated ethnicity) prior to administration of primaquine for radical treatment of *P. vivax* infection, and also shed further light on the evolutionary history of G6PD variants in humans.

## References

[1] Cappellini MD, Fiorelli G (2008) Glucose-6-phosphate dehydrogenase deficiency. Lancet 371: 64–74. 18177777 10.1016/S0140-6736(08)60073-2

[2] Cocco P, Todde P, Fornera S, Manca MB, Manca P, et al (1998) Mortality in a cohort of men expressing the glucose-6-phosphate dehydrogenase deficiency. Blood 91: 706–709. 9427729

[3] WHO Working Group (1989) Glucose-6-phosphate dehydrogenase deficiency. Bull World Health Organ. 601–611. PMC2491315 2633878

[4] Beutler E, Yeh M, Fairbanks VF (1962) The normal human female as a mosaic of X-chromosome activity: studies using the gene for G-6-PD-deficiency as a marker. Proc Natl Acad Sci U S A 48: 9–16. 13868717 10.1073/pnas.48.1.9 PMC285481

[5] Liu WL, Li F, He ZX, Jiang HY, Ai R (2013) Identification of a case of glucose-6-phosphate dehydrogenase deficiency with G6PD mediterranean-middle east subtype in China. Int J Lab Hematol 35: e1–3. 10.1111/ijlh.12046 23286329

[6] Ainoon O, Yu YH, Amir Muhriz AL, Boo NY, Cheong SK, et al (2003) Glucose-6-phosphate dehydrogenase (G6PD) variants in Malaysian Malays. Hum Mutat 21: 101. 10.1002/humu.9103 12497642

[7] Saha S, Saha N, Tay JS, Jeyaseelan K, Basair JB, et al (1994) Molecular characterisation of red cell glucose-6-phosphate dehydrogenase deficiency in Singapore Chinese. Am J Hematol 47: 273–277. 7977299 10.1002/ajh.2830470405

[8] WHO (2010) Guidelines for the treatment of malaria (2nd edition): WHO.

[9] Moiz B, Nasir A, Moatter T, Naqvi ZA, Khurshid M (2011) Molecular characterization of glucose-6-phosphate dehydrogenase deficiency in Pakistani population. Int J Lab Hematol 33: 570–578. 21507207 10.1111/j.1751-553X.2011.01325.x

[10] Saha N, Ramzan M, Tay JS, Low PS, Basair JB, et al (1994) Molecular characterisation of red cell glucose-6-phosphate dehydrogenase deficiency in north-west Pakistan. Hum Hered 44: 85–89. 8188314 10.1159/000154196

[11] Leslie T, Briceño M, Mayan I, Mohammed N, Klinkenberg E, et al (2010) The impact of phenotypic and genotypic G6PD deficiency on risk of Plasmodium vivax infection: a case-control study amongst Afghan refugees in Pakistan. PLoS Med 7: e1000283. 20520804 10.1371/journal.pmed.1000283 PMC2876136

[12] Rebholz CE, Michel AJ, Maselli DA, Saipphudin K, Wyss K (2006) Frequency of malaria and glucose-6-phosphate dehydrogenase deficiency in Tajikistan. Malar J 5: 51. 16780577 10.1186/1475-2875-5-51 PMC1533840

[13] Bakhramov SM, Ashrabhodzhaeva KK (2011) [Erythrocytic enzymopathy in Uzbekistan]. Lik Sprava: 73–77. 22768742

[14] WHO-EMRO (2012) World Malaria Report, Afghanistan. WHO.

[15] Heller P, Best WR, Nelson RB, Becktel J (1979) Clinical implications of sickle-cell trait and glucose-6-phosphate dehydrogenase deficiency in hospitalized black male patients. N Engl J Med 300: 1001–1005. 431593 10.1056/NEJM197905033001801

[16] Hoiberg A, Ernst J, Uddin DE (1981) Sickle cell trait and glucose-6-phosphate dehydrogenase deficiency. Effects on health and military performance in black Navy enlistees. Arch Intern Med 141: 1485–1488. 7283560

[17] Samilchuk E, D’Souza B, Al-Awadi S (1999) Population study of common glucose-6-phosphate dehydrogenase mutations in Kuwait. Hum Hered 49: 41–44. 9858856 10.1159/000022838

[18] Kurdi-Haidar B, Mason PJ, Berrebi A, Ankra-Badu G, al-Ali A, et al (1990) Origin and spread of the glucose-6-phosphate dehydrogenase variant (G6PD-Mediterranean) in the Middle East. Am J Hum Genet 47: 1013–1019. 1978555 PMC1683892

[19] Vulliamy TJ, Othman A, Town M, Nathwani A, Falusi AG, et al (1991) Polymorphic sites in the African population detected by sequence analysis of the glucose-6-phosphate dehydrogenase gene outline the evolution of the variants A and A. Proc Natl Acad Sci U S A. 88: 8568–8571. 10.1073/pnas.88.19.8568 PMC52550 1924316

[20] Tang TK, Huang CS, Huang MJ, Tam KB, Yeh CH, et al (1992) Diverse point mutations result in glucose-6-phosphate dehydrogenase (G6PD) polymorphism in Taiwan. Blood 79: 2135–2140. 1562739

[21] Project G (2010) A map of human genome variation from population-scale sequencing. Nature 467: 1061–1073. 20981092 10.1038/nature09534 PMC3042601

[22] Beutler E, Kuhl W (1990) The NT 1311 polymorphism of G6PD: G6PD Mediterranean mutation may have originated independently in Europe and Asia. Am J Hum Genet 47: 1008–1012. 1978554 PMC1683912

[23] Genomes Project (2012) An integrated map of genetic variation from 1,092 human genomes. Nature 491: 56–65. 23128226 10.1038/nature11632 PMC3498066

[24] Sarkar S, Biswas NK, Dey B, Mukhopadhyay D, Majumder PP (2010) A large, systematic molecular-genetic study of G6PD in Indian populations identifies a new non-synonymous variant and supports recent positive selection. Infect Genet Evol 10: 1228–1236. 20713184 10.1016/j.meegid.2010.08.003

[25] Sirdah M, Reading NS, Perkins SL, Shubair M, Aboud L, et al (2012) Hemolysis and Mediterranean G6PD mutation (c.563 C>T) and c.1311 C>T polymorphism among Palestinians at Gaza Strip. Blood Cells Mol Dis 48: 203–208. 22364808 10.1016/j.bcmd.2012.01.007

[26] Al-Allawi N, Eissa AA, Jubrael JM, Jamal SA, Hamamy H (2010) Prevalence and molecular characterization of Glucose-6-Phosphate dehydrogenase deficient variants among the Kurdish population of Northern Iraq. BMC Blood Disord 10: 6. 20602793 10.1186/1471-2326-10-6 PMC2913952

[27] Al-Musawi BM, Al-Allawi N, Abdul-Majeed BA, Eissa AA, Jubrael JM, et al (2012) Molecular characterization of glucose-6-phosphate dehydrogenase deficient variants in Baghdad city - Iraq. BMC Blood Disord 12: 4. 22452742 10.1186/1471-2326-12-4 PMC3323424

[28] Rahimi Z, Vaisi-Raygani A, Nagel RL, Muniz A (2006) Molecular characterization of glucose-6-phosphate dehydrogenase deficiency in the Kurdish population of Western Iran. Blood Cells Mol Dis 37: 91–94. 16938474 10.1016/j.bcmd.2006.07.004

[29] Chalvam R, Colah RB, Mohanty D, Ghosh K, Mukherjee MB (2011) Restriction fragment length polymorphism (RFLP) of the X chromosome linked glucose-6-phosphate dehydrogenase (G6PD) locus in India. Ann Hum Biol 38: 106–109. 20528626 10.3109/03014460.2010.488251

[30] Moiz B, Nasir A, Moatter T, Naqvi ZA, Khurshid M (2009) Population study of 1311 C/T polymorphism of Glucose 6 Phosphate Dehydrogenase gene in Pakistan - an analysis of 715 X-chromosomes. BMC Genet 10: 41. 19640310 10.1186/1471-2156-10-41 PMC2725355

[31] Sukumar S, Mukherjee MB, Colah RB, Mohanty D (2004) Molecular basis of G6PD deficiency in India. Blood Cells Mol Dis 33: 141–145. 15315792 10.1016/j.bcmd.2004.06.003

[32] Leslie T, Moiz B, Mohammad N, Amanzai O, Ur Rasheed H, et al (2013) Prevalence and molecular basis of glucose-6-phosphate dehydrogenase deficiency in Afghan populations: implications for treatment policy in the region. Malar J 12: 230. 23834949 10.1186/1475-2875-12-230 PMC3710480

[33] Bouma MJ, Goris M, Akhtar T, Khan N, Khan N, et al (1995) Prevalence and clinical presentation of glucose-6-phosphate dehydrogenase deficiency in Pakistani Pathan and Afghan refugee communities in Pakistan; implications for the use of primaquine in regional malaria control programmes. Trans R Soc Trop Med Hyg 89: 62–64. 7747310 10.1016/0035-9203(95)90661-4

[34] Tishkoff SA, Varkonyi R, Cahinhinan N, Abbes S, Argyropoulos G, et al (2001) Haplotype diversity and linkage disequilibrium at human G6PD: recent origin of alleles that confer malarial resistance. Science 293: 455–462. 11423617 10.1126/science.1061573

[35] Sabeti PC, Reich DE, Higgins JM, Levine HZ, Richter DJ, et al (2002) Detecting recent positive selection in the human genome from haplotype structure. Nature 419: 832–837. 12397357 10.1038/nature01140

[36] Haber M, Platt DE, Ashrafian Bonab M, Youhanna SC, Soria-Hernanz DF, et al (2012) Afghanistan’s ethnic groups share a Y-chromosomal heritage structured by historical events. PLoS One 7: e34288. 22470552 10.1371/journal.pone.0034288 PMC3314501

[37] Louicharoen C, Patin E, Paul R, Nuchprayoon I, Witoonpanich B, et al (2009) Positively selected G6PD-Mahidol mutation reduces Plasmodium vivax density in Southeast Asians. Science 326: 1546–1549. 20007901 10.1126/science.1178849

[38] Clark TG, Fry AE, Auburn S, Campino S, Diakite M, et al (2009) Allelic heterogeneity of G6PD deficiency in West Africa and severe malaria susceptibility. Eur J Hum Genet 17: 1080–1085. 19223928 10.1038/ejhg.2009.8 PMC2986558

